# Isotretinoin Exposure and Risk of Celiac Disease

**DOI:** 10.1371/journal.pone.0135881

**Published:** 2015-08-19

**Authors:** Shadi Rashtak, Shahryar Khaleghi, Eric V. Marietta, Mark R. Pittelkow, Joseph J. Larson, Brian D. Lahr, Joseph A. Murray

**Affiliations:** 1 Department of Dermatology, Mayo Clinic, Rochester, Minnesota, United States of America; 2 Department of Gastroenterology and Hepatology, Mayo Clinic, Rochester, Minnesota, United States of America; 3 Department of Immunology, Mayo Clinic, Rochester, Minnesota, United States of America; 4 Department of Biomedical Statistics and Informatics, Mayo Clinic, Rochester, Minnesota, United States of America; Harvard Medical School, UNITED STATES

## Abstract

**Background:**

Isotretinoin (13-cis retinoic acid) is a metabolite of vitamin A and has anti-inflammatory and immunoregulatory effects; however, a recent publication by DePaolo et al. demonstrated that in the presence of IL-15, retinoic acid can act as an adjuvant and promote inflammation against dietary proteins.

**Objective:**

To evaluate the risk of overt and latent celiac disease (CD) among users of isotretinoin.

**Material and Methods:**

Medical records of patients from 1995 to 2011 who had a mention of isotretinoin in their records (N = 8393) were searched for CD diagnosis using ICD-09CM codes. Isotretinoin exposure was compared across overt CD patients and their age- and gender-matched controls from the same pool. To evaluate the risk of latent CD with isotretinoin exposure, patients were overlapped with a community-based list of patients with waste serum samples that were tested for CD serology, excluding those with overt CD (2006–2011). Isotretinoin exposure was defined as the use of isotretinoin prior to CD diagnosis or serology.

**Results:**

Of 8393 patients, 25 had a confirmed CD diagnosis. Compared to matched controls (N = 75), isotretinoin exposure was not significantly different between overt CD patients versus controls (36% versus 39%, respectively; P = 0.712). Likewise, latent CD defined as positive serology was not statistically different between isotretinoin exposed (N = 506) versus non-exposed (N = 571) groups (1.8% versus 1.4%, respectively; P = 0.474).

**Conclusions:**

There was no association between isotretinoin use and risk of either overt or latent CD.

## Introduction

Celiac disease (CD) is an immune-mediated disease of the small intestine that develops upon exposure to oral gluten in genetically susceptible individuals (HLA-DQ2 or -DQ8 carriers). Isotretinoin (also known as 13-cis-retinoic acid) is a synthetic form of vitamin A, which is primarily used to treat severe nodulocystic acne that fails to respond to other treatments such as oral antibiotics. Isotretinoin regulates cell proliferation and differentiation by activating nuclear retinoic acid receptors.

There is controversy in the literature as to whether isotretinoin exposure could be linked to inflammatory intestinal diseases. While one case-control study has found an association between isotretinoin exposure and development of ulcerative colitis [1] the majority of reports fail to demonstrate a link between the two [2,3,4,5].

In addition to the contrary human studies, in vitro and animal studies also show conflicting results regarding the role of retinoic acid on the inflammatory response in the gut. In contrast to the previous studies that showed retinoic acid inhibited interleukin (IL)-6 driven proinflammatory responses and induced the generation of regulatory T cells (Tregs) [6,7] a recent study demonstrated that in the presence of IL-15, retinoic acid promoted an inflammatory T cell response to dietary antigens in DQ8-Dd-IL-15 mice, mimicking early stage CD [8].

In the present study, we aimed to evaluate the association between prior isotretinoin use and CD. In the first part of the study, we identified diagnosed CD cases, and compared their history of isotretinoin use to that in age- and sex-matched controls. To study the effect of exposure on undiagnosed CD, we identified the subset of patients with a mention of isotretinoin in the records that overlapped with a community-based cohort of tested waste serum samples and tested the association between isotretinoin exposure and positive CD serology.

## Materials and Methods

### Patients

The Mayo Clinic Institutional Review Board approved the study. The underlying population that we studied was patients who had a mention of isotretinoin or its brand names in their medical records. This population would include those patients who were exposed to isotretinoin as well as others who were considered for it but ultimately not exposed to the drug. This inclusion criterion likely selected a homogenous group of patients and minimized imbalances between those treated and untreated in terms of history of acne or prior antibiotic use. To identify the patients who had a mention of isotretinoin in their records, we used the Data Discovery and Query Builder (DDQB) toolset. Using DDQB, the electronic medical records of patients who were seen at Mayo Clinic Rochester, Minnesota from June 01, 1995 to June 1, 2011 were searched for the terms “isotretinoin” and its brand names “Accutane”, “Sotret”, “Claravis”, and “Amnesteem”. We identified a total of 8393 patients for whom isotretinoin was referenced in their medical records.

**Fig 1:**
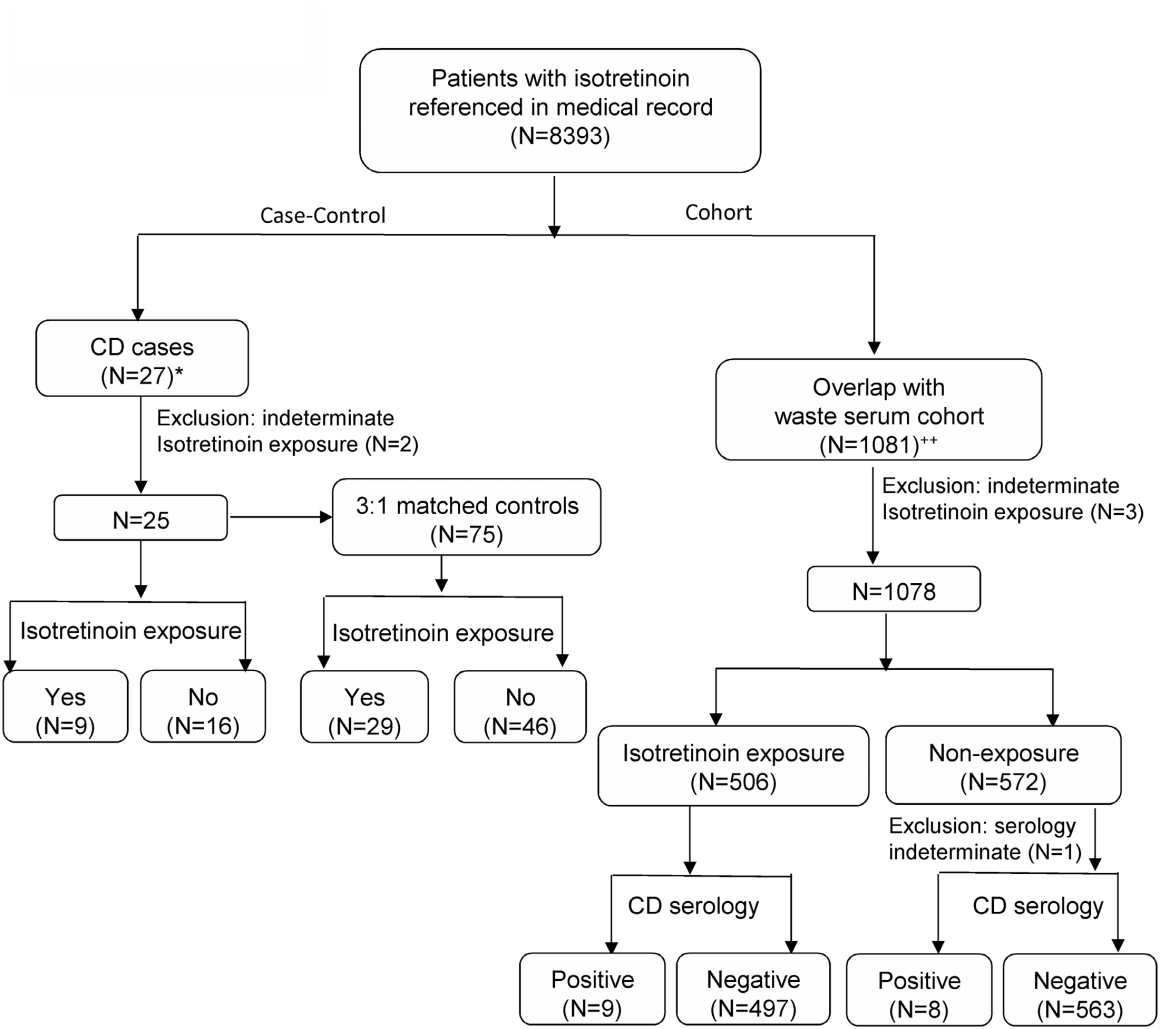
Flow diagram showing the number of patients who met the inclusion and exclusion criteria in each arm. In the left arm isotretinoin exposure was assessed among diagnosed CD patients and their age- and gender-matched controls (case-control design). In the right arm, latent CD (defined as positive CD serology) was assessed among patients with and without isotretinoin exposure (cohort design).

In the first part of the study, we aimed to assess if the exposure to isotretinoin was different between diagnosed CD cases and controls. To identify CD patients, we searched ICD-09CM codes for CD diagnosis (ICD-09CM code = 579.0). A total of 63 patients out of 8393 patients were identified who were coded with CD at some point during their medical care at the Mayo Clinic. The medical records of these 63 patients were then carefully reviewed and then cross-referenced with a comprehensive CD registry database for purposes of verification. Out of 63 patients, 34 patients were identified as not having CD after evaluation and an additional two patients were excluded as their true CD status could not be determined. The remaining 27 patients had a true diagnosis of CD based on positive intestinal biopsies, though 2 of these cases had unknown isotretinoin exposure and were thus excluded. For each of the 25 cases with diagnosed CD, we selected 3 age- and gender-matched controls from the pool of patients who had a mention of isotretinoin in their records. Isotretinoin exposure was then abstracted from the medical records for both cases and controls. Exposure was defined as a history of taking isotretinoin prior to the diagnosis of CD (date of intestinal biopsy) for cases and the corresponding dates for matched controls (Fig 1). In the second part of the study, we explored the relationship between isotretinoin use and latent CD in a large community-based convenience sample. In particular, the set of patients who had some reference to isotretinoin in their records was merged with an existing cohort of tested waste serum, which excluded those with a previous diagnosis of CD. Between June 2006 and June 2011, we used waste serum samples of adults collected in a previous study of ours, in which individuals between the ages of 18 to 50 had a blood test done for any reason.

To avoid the referral bias of a tertiary medical center, serum samples were only collected from patients who were residents of Olmsted County at the time of blood test. For the overlapping group between the two lists (n = 1081), the medical record of each patient was reviewed manually. Data on patient demographics, isotretinoin exposure, indication for isotretinoin treatment consideration, and antibiotic and tetracycline use were abstracted. Patients who had an isotretinoin exposure prior to serum sample collection were placed into the isotretinoin-exposed group, and those who never had isotretinoin or used it after serum sample collection were placed into the non-exposed group. We excluded three patients due to indeterminate isotretinoin exposure and one patient due to indeterminate serology, leaving a total of 1077 patients used in the final analysis (Fig 1). Exposure to systemic antibiotics or systemic tetracyclines (i.e. tetracycline, doxycycline, minocycline, etc) was defined as history of using these medications for the purpose of acne treatment. For many patients who had a remote history of acne or isotretinoin use, there tended to be little to no information on antibiotic treatment in the medical record. We assumed this absence of data reflected no history of antibiotics, and thus these patients were grouped along with those who were known to have not taken antibiotics for acne in the analysis.

### Celiac Disease Serology

The waste serum samples had been tested for tissue transglutaminase immunoglobulin A (TTG-IgA) using an enzyme-linked immunosorbent assay as the initial screening (INOVA Diagnostics, Inc, San Diego, CA) [9]. Test results were considered negative if TTG-IgA level were less than 2.0 U/ml. Samples with TTG-IgA levels greater than 2.0 were subsequently tested for the presence of endomysial antibodies (EMA) using an immunofluorescence assay (Beckman Coulter, Inc, Brea, CA). The result was considered positive if fluorescence was observed at a dilution of 1:≥5. Latent CD was defined by positive serology based on the presence of a TTG-IgA level greater than 2.0 U/mL accompanied with a positive EMA test. Serology was considered to be negative if the TTG-IgA level was less than 2.0 U/mL or if it was between 2.0 and 4.0 U/mL and accompanied with a negative EMA result; the serum was considered to be indeterminate if TTG-IgA level was greater than 4.0 U/mL and the EMA test was negative. The technologist reading the EMA assay was blinded to the TTG-IgA status.

### Statistical Analysis

Descriptive statistics are presented to summarize patient characteristics: mean and standard deviation (SD) for continuous variables, or counts and percentages for categorical variables. Two group comparisons were formally evaluated using standard tests of association (Student’s t-test, chi-square test or Fisher’s exact test, as appropriate). In observational studies, a propensity score approach can be useful for adjusting for confounding from nonrandom treatment assignment through data reduction, particularly when the patient outcome of interest is rare such that power is insufficient for standard covariate adjustment [10]. We estimated the propensity score as the predicted value (logit-transformed predicted probability) from a multivariable logistic model where the response variable was isotretinoin status and the explanatory variables were the observable covariates possibly influencing treatment selection: age, gender, race, indication of visit, and prior antibiotic use. In a second step the estimated propensity score was included as a single adjustment variable, along with the main effect of isotretinoin exposure, in a logistic model with overt CD (diagnosed CD case vs. control) as the target of prediction. These steps were then repeated in the overlapping set with waste serum to assess the effect of isotretinoin exposure on the outcome of latent CD (positive vs. negative serology). All analyses were performed using SAS statistical software package (version 9.3, SAS Institute, Cary, NC). A type I error rate of 0.05 was used to determine statistical significance.

## Results

### Isotretinoin-Exposure and Risk of Overt Celiac Disease

The first part of this study utilized a case-control design to evaluate if the exposure to isotretinoin is associated with a subsequent diagnosis of CD. From the pool of patients who had a reference to isotretinoin in their medical records, we identified 25 overt CD cases and compared these to 3 to 1 age- and sex-matched control (Table 1). In addition to the matching variables, both groups were similar in race (predominately Caucasian) and indication for discussion/consideration of isotretinoin (acne being the main reason in the majority). Based on an unadjusted comparison, isotretinoin exposure was not significantly different among CD cases versus controls (36.0% versus 38.7%, respectively; P = 0.812). Adjusted for the five baseline covariates in a propensity score, there was no significant association between prior isotretinoin exposure and CD case status (P = 0.712).

**Table 1:** Comparins Overt CD Cases and Controls.

Variable		CD Case (n = 25)	Control (n = 75)	P-value
Age at CD Assessment^‡^		30.6±13.2	30.6±12.9	0.994
Female		21 (84.0%)	63 (84.0%)	1.000
Race:				1.000*
	Non-White	0 (0.0%)	2 (2.7%)	
	White	24 (96.0%)	69 (92.0%)	
	Unknown Race	1 (4.0%)	4 (5.3%)	
Indication:				0.806*
	Non-Acne	5 (20.0%)	13 (17.3%)	
	Acne	19 (76.0%)	60 (80.0%)	
	Unknown Indication	1 (4.0%)	2 (2.7%)	
Systemic Antibiotics Use:				0.697*
	None	14 (56.0%)	38 (50.7%)	
	Other Med	1 (4.0%)	2 (2.7%)	
	Tetracycline	10 (40.0%)	35 (46.7%)	
Isotretinoin Exposure		9 (36.0%)	29 (38.7%)	0.812^†^

Unless noted otherwise, data summarized with counts (%) and compared between groups using a Chi-square test

*Groups compared using Fisher's exact test due to sparse data

^‡^Age distribution summarized with mean±SD and evaluated for a group difference using a two sample t test

^†^P-value = 0.712 from logistic regression model adjusting for propensity score.

### Isotretinoin-Exposure and Risk of Latent Celiac Disease

In the second part of the study based on a convenience sample with tested waste serum, we assessed the frequency of latent (undiagnosed) CD, defined as positive serology, among patients with and without isotretinoin exposure. The final analysis set was comprised of patients who 1) had a reference to isotretinoin in their records, 2) had a tested waste serum between June 2006 and June 2011, and 3) were Olmsted county residents between the ages of 18 to 50. Excluding one subject with an indeterminate serology result, there were a total of 1077 subjects, 506 who had exposure to isotretinoin before their serum sample collection and 571 who did not. Table 2 shows the characteristics of both exposed and non-exposed groups. There were statistically significant differences in age, gender, indication for isotretinoin treatment/consideration, antibiotic use and tetracycline use between the two groups. From an unadjusted analysis the proportion with latent CD was not significantly different between groups (1.8% in isotretinoin-exposed versus 1.4% in non-exposed; P = 0.620). Using regression adjustment for the propensity score to control for baseline differences in the treatment groups, the association between isotretinoin exposure and latent CD remained insignificant (P = 0.474)

**Table 2:** Isotretinoin Exposure and Latent CD.

Variable		Exposed (n = 506)	Unexposed (n = 571)	P-value
Age at Serum Collection^‡^		31.0±8.7	29.0±8.9	<.001
Female		308 (60.9%)	404 (70.8%)	<.001
Race:				0.311
	Non-White	32 (6.3%)	48 (8.4%)	
	White	452 (89.3%)	504 (88.3%)	
	Unknown Race	22 (4.3%)	19 (3.3%)	
Indication:				<.001
	Non-Acne	17 (3.4%)	47 (8.2%)	
	Acne	489 (96.6%)	524 (91.8%)	
Systemic Antibiotics Use:				0.040
	None	151 (29.8%)	132 (23.1%)	
	Other Med	28 (5.5%)	38 (6.7%)	
	Tetracycline	327 (64.6%)	401 (70.2%)	
Positive Serology		9 (1.8%)	8 (1.4%)	0.474^†^

Unless noted otherwise, data summarized with counts (%) and compared between groups using a Chi-square test

^‡^Age distribution summarized with mean+SD and evaluated for a group difference using a two sample t test

^†^P-value from logistic regression model adjusting for propensity score.

## Discussion

To our knowledge, this is the first study to evaluate the relationship between isotretinoin exposure and risk of overt and latent CD. Our results did not show a significant association of either overt CD or latent CD based on exposure to isotretinoin. Our results are consistent with the majority of the studies that have evaluated the effect of isotretinoin exposure on other inflammatory diseases of the intestines such as Crohn’s disease and ulcerative colitis[2,3,4].

The effect of retinoic acid on the inflammatory response in the gut is quite complex and involves multiple molecules and pathways. While some studies suggest an anti-inflammatory and immunomodulatory effect for retinoic acid in the gut [6,11,12,13] others show that in some settings such as over expression of IL-15, retinoic acid could exert an inflammatory role upon the intestine [8].

Regulatory T cells (Tregs) in the intestine are important factors in achieving immune tolerance to dietary antigens. It is shown that dendritic cells of mesenteric lymph nodes and lamina propria induce intestinal Tregs through a retinoic acid-dependent mechanism [11,14]. Other than its key role in promoting Tregs, retinoic acid also inhibits IL-6 dependent generation of pro-inflammatory T helper (TH)-17 cells[6]. Moreover, retinoic acid in the forms of all-trans retinoic acid and 9-cis retinoic acid is shown to suppress TH1 cells and produce development of TH2 cells, and in this manner exert its immunomodulatory effect [12]. Given all these properties, one might speculate that retinoic acid should have potentially protecting effects in regards to CD, which is thought to be a primarily TH1-mediated inflammatory disorder [15]. Nonetheless, in a recent study, DePaolo *et al* showed that in the setting of high levels of IL-15 in the intestine, retinoic acid acts on dendritic cells promoting secretion of IL-12 and IL-23 pro-inflammatory cytokines leading to induction of TH1 and TH17 cells, respectively [8]. In their genetically engineered mouse model of CD (DQ8-Dd-IL-15) which carries the CD susceptibility gene (HLA-DQ8), and constitutively produces high levels of IL-15 in the intestine, retinoic acid promotes a TH1 inflammatory response against dietary gluten, mimicking early stage CD [8].

Although the association between isotretinoin exposure and CD simply may not exist, several other factors might also explain why a true association was not found in our study. First of all, we were not able to address the levels of IL-15 expression in the lamina propria of overt and latent CD patients in our study. While upregulation of IL-15 in the intestine of CD patients is well-documented [16,17], the study by Depaolo *et al* shows that these levels vary greatly in patients with active CD [8]. It is possible that the levels of lamina propria IL-15 expression need to be constitutively high in patients at risk for developing CD in order for exogenous retinoic acid (such as isotretinoin) to exert an inflammatory role upon the intestine. Moreover, given the counteracting effects of retinoic acid on prevention or promotion of immunity against dietary antigens depends on certain immunologic context, variation in those settings among CD susceptible individuals can result in loss of any association between isotretinoin exposure and CD. Finally, because statistical power in this type of analysis is primarily determined by the number of patients who had the outcome, the small number of overt and latent CD patients (despite the fairly large number of total patients) could potentially mean our study was underpowered to detect an association.

The prevalence of CD ranges from 0.1% to 2.7% across different countries [18]. In this study, we found a proportion of 17/1077 (1.6%) for latent CD among our population of mainly acne patients who were considered for isotretinoin treatment. This may be slightly higher than the reported prevalence of 1.0% among non-Hispanic whites in the United States [19], though our sample was generally younger (average age about 30 years) and nearly two-thirds female. It is also possible that long-term antibiotic use in acne patients could have a potential role in increasing the risk of CD as compared to the general population. Intestinal microbiota has an important role in the development of intestinal immune system and establishment of oral tolerance [20,21]. Changes in intestinal microbiota have been implicated in the pathogenesis of CD [22,23]. As such antibiotics can potentially have a role in the pathogenesis of CD through their impact on the intestinal microbiota. In fact, a recent case-control study has shown that systemic antibiotic use is associated with increased risk of CD [24].

In this study, the majority of patients in both isotretinoin-exposed and non-exposed group received systemic antibiotics for acne. In about 30% of patients, there was no mention of antibiotic use in their medical record, and thus we assumed these were patients without a history of long-term antibiotic use. Nonetheless, it is quite possible that some of these patients lacking these notes had actually received systemic antibiotics, since isotretinoin is not usually the first-line treatment for acne.

The strengths of our study include its novelty as well as its community-based design which has allowed us to evaluate the association between isotretinoin and risk of both overt and latent CD for the first time. In addition, the selection criteria of including only patients who had a mention of isotretinoin in their records likely reduced confounding factors between the treated and untreated groups. In addition, we adjusted for residual confounding variables in a propensity score while measuring the exposure effect on both CD outcomes. Also by selecting the patients from a pool of community-based convenience sample, we were able to avoid the potential selection bias of a tertiary medical center and report more generalizable results.

There are some limitations of the study though. One would be the small numbers of overt and latent CD cases and another is the retrospective design. Moreover, due to questionable precision of ascertaining data on systemic antibiotic use, we did not test for the association between antibiotic use and CD in our study. In addition, the length of exposure to isotretinoin assayed in this study may not have been long enough to increase the risk of CD. The normal dosage of isotretinoin for these patients is between 0.5 mg to 1 mg/ kg/day, resulting in 120 to 150 mg/kg for a cumulative dose over 5 months, which was the time period that the majority of the patients took isotretinoin.

In the DePaolo et al mouse study, retinoic acid (1uM) given every other day for 10 days increased the inflammatory responses to Ova; however, it is not clear what dose or duration of isotretinoin therapy could potentially trigger CD in human, if any. It appears though that the routine doses and lengths of treatment used in isotretinoin therapy does not increase risk of CD among patients.

Further studies with larger sample size are needed to confirm these results with certainty and to address other potential confounding factors when evaluating this relationship.
